# Electroacupuncture therapy for change of pain in classical trigeminal neuralgia

**DOI:** 10.1097/MD.0000000000019710

**Published:** 2020-04-17

**Authors:** Jing Sun, Rongrong Li, Xiaoyu Li, Lifang Chen, Yi Liang, Qifei Zhang, Ruohan Sun, Hantong Hu, Xiaomei Shao, Jianqiao Fang

**Affiliations:** a The Third Clinical Medical College, Zhejiang Chinese Medical University, Key Laboratory of Acupuncture and Neurology of Zhejiang Province; b The Third Affiliated Hospital of Zhejiang Chinese Medical University, Hangzhou, Zhejiang, China.

**Keywords:** change of pain, classical trigeminal neuralgia, electroacupuncture, protocol, randomized clinical trial

## Abstract

**Introduction:**

Classical trigeminal neuralgia (CTN) is a kind of trigeminal neuralgia which is due to neurovascular compression. The common neurological treatment CTN drug called carbamazepine is the main measure, although it usually has side effects and a high-rate of relapse. As a critical alternative therapy, electroacupuncture (EA) has been shown to benefit for neuropathic pain. The aims of this study are to observe the therapeutic effect and safety of EA for CTN, to evaluate whether EA has the advantage over carbamazepine in the analgesia of CTN. Furthermore, we would to establish a standardized, effective, and convenient therapy program of EA.

**Methods and analysis:**

One hundred twenty patients diagnosed with CTN will be randomized for a 4-week intervention. The interventions will be different according to the four groups (EA + carbamazepine group, sham EA + carbamazepine group, EA + placebo group and sham EA + placebo group). EA therapy will be performed in specific acupoints with a dilute wave (2/100 Hz) for 60 minutes. Carbamazepine tablets will be taken orally with 0.1 g each time, thrice daily. Sham EA and placebo intervention will not receive EA and drug treatment. The main outcomes are the change from baseline intensity of pain at 6 months (pain evaluation by visual analogue score) and the change from baseline brief introduction of 2-week pain to evaluate pain comprehensively. The data management and statistical analysis will be conducted by third party statisticians. Incidence of adverse events will be investigated.

**Ethics and dissemination:**

Ethics approval was obtained from the Clinical Trial Ethics Committee of The Third Affiliated Hospital of Zhejiang Chinese Medical University (NO. ZSLL-KY-2017-033) and Jiaxing Hospital of Traditional Chinese Medicine (NO. 2018-JZLK-002). The results will be disseminated by presentation at peer-reviewed journals.

## 1 Introduction

Trigeminal neuralgia is a common form of facial neuropathic pain. It is classified as classical trigeminal neuralgia (CTN) and painful trigeminal neuralgia, which are also called “idiopathic” and “secondary” in usual. CTN includes all cases without an established etiology.^[1]^ The diagnosis of CTN also requires that there be no clinically evident neurologic deficit except neurovascular compression. It is a chronic pain characterized by brief electric shock-like, shooting, stabbing pains in ≥1 branches of the trigeminal nerve,^[2]^ and repeat in paroxysmal attacks lasting from a fraction of 1 second to 2 minutes of one side of face. The disease severely affects the quality of life and causes some emotional disturbances, such as anxiety, depression and insomnia.^[3]^ It also affects lifestyle due to the common activities such as eating, chewing, talking, washing the face, brushing the teeth, which may trigger the pain. It is estimated that approximately 4.3 to 8 per 100000 people worldwide suffered from CTN, and the number affected tends to be higher among women at all ages; the average age of mobility is 47 to 79 years.^[4–6]^

According to the criteria of European Federation of Neurological Societies (EFNS) 2010,^[1]^ the effective drug for treatment of CTN is carbamazepine,^[7,8]^ which can reduce both the frequency and intensity of painful paroxysmal. However, the efficiency will be suppressed because of poorly tolerated, obvious gastrointestinal reaction and liver toxicity, adverse events with anemia, thrombocytopenia, and leukopenia.^[1,9–11]^ Surgical treatments including microvascular decompression, pulsed radio frequency, gamma knife surgery, and percutaneous micro-balloon compression are also available for CTN.^[12,13]^ But some studies have pointed out limitations including the recurrence after surgery, postoperative complications such as facial sensation disorders, chewing weakness, facial paralysis, and hearing impairment.^[14,15]^ In addition, in couple with the patient's psychological fear of surgery, it was also not appropriate to treat CTN.

Acupuncture originated >3000 years ago in China and has gained popularity in the United States since the late 1970s.^[16]^ Over its development, a wealth of experience has accumulated in the practice of acupuncture for the chronic disease and pain control. There is evidence that acupuncture is favorable in treating neuropathic pain.^[17]^ As the development of acupuncture, electroacupuncture (EA) has a clear advantage in the treatment of trigeminal neuralgia.^[18–20]^ EA can inhibit the generation of pain sensation, improve hemodynamics, and achieve positive and negative effects of blood pressure to reduce the compression of blood vessels.^[21]^ EA has a certain clinical effect on the treatment of CTN, whereas there are few reports with a proper methodology in literature.^[22]^ The original studies had exposed limitations according to the evidence-based medicine research. It is necessary to carry out a high-quality, well-designed randomized controlled clinical trial.

This clinical trial will recruit enough CTN patients to observe the therapeutic effect and safety of EA for CTN, to evaluate whether EA has the advantage over carbamazepine in the analgesia of CTN. Furthermore, we would to establish a standardized, effective and convenient therapy program of EA to promote it widely.

## 2 Methods and analysis

### 2.1 Study design

**Figure 1:**
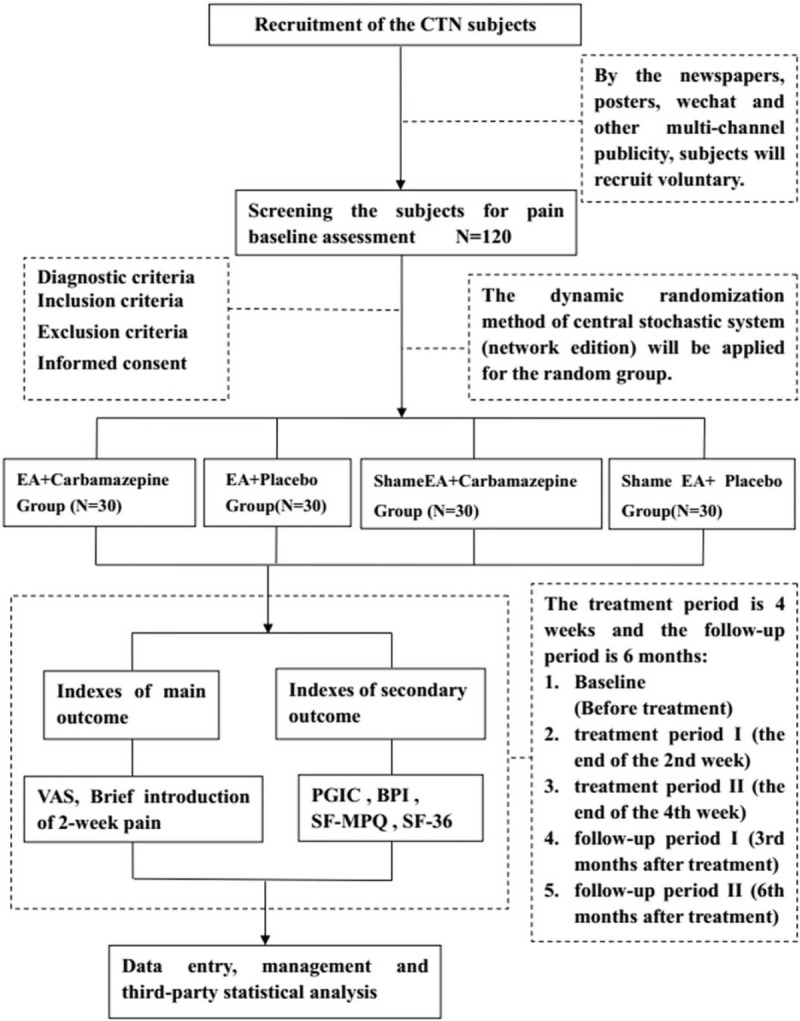
Technical route of study design. CTN = classical trigeminal neuralgia, EA = electroacupuncture, VAS = visual analogue score, PGIC = patient global impression of change, BPI = brief pain inventory-facial scale, SF-MPQ = short-form McGill pain questionnaire, SF-36 = short- form 36 questionnaire).

This is a 2-center, randomized, sham-controlled, placebo-controlled, participant-blinded, and assessor-blinded, exploratory clinical trial. Participants recruited from 2 large Chinese medical hospitals will be randomly divided into 4 groups (EA + carbamazepine group, sham EA + carbamazepine group, EA + placebo group, and sham EA + placebo group) by applying dynamic randomization method of central stochastic system. The study participants’ flow outline and trial schedule are shown in Figure 1 and Table 1. The eligibility criteria are fairly well established. Any change in the criteria or methodology will be communicated to the entire research team in a conference. All changes will be included in the final write-up for a journal submission. We used the SPIRIT checklist when writing our report.

**Table 1:**
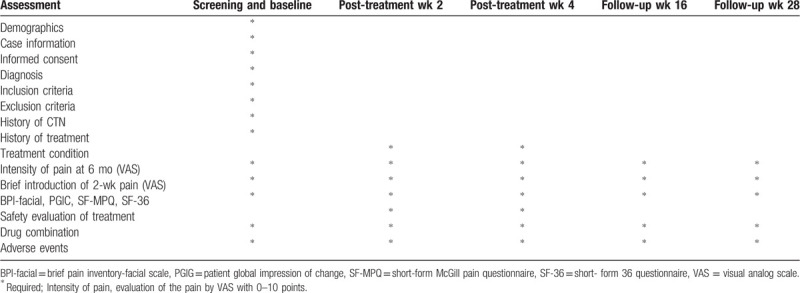
Trial schedule.

### 2.2 Study methods

#### 2.2.1 Sample size estimation

The SAS software, V9.3, is applied to estimate the sample size. According to the literature and the preliminary experiment, the mean values of the visual analog scale (VAS) score of EA + Carbamazepine group, EA + Placebo group, sham EA + Carbamazepine group, sham EA + Placebo group ware 5.23, 4.45, 5.50, 0.00. The standard deviation was 1.6. Under the condition of α = 0.05, the test efficiency 1-β = 0.8, the 4 groups were allocated with the 1:1:1:1 proportion; the sample size of each group was calculated at least 24. Considering the existence of shedding and other factors, in the premise of guaranteeing the minimum sample size, the sample size was enlarged by 20% to 30% cases in 1 group, in total 120 cases for 4 groups. Because of the complexity of the sample calculation process of factorial design, the formula is not listed here.

#### 2.2.2 Participants recruitment and ethics

The recruiting time is from July 1, 2018 to December 31, 2021. The time of first patient enrolment was after the registration of trial. This trial had been registered at ClinicalTrials.gov (NO. NCT03580317, 06/25/2018). Receptors of this trial will be recruited by putting up posters in hospitals and advertising in the health-related internet, WeChat, local newspapers in Hangzhou city. After evaluation and filtration by applying diagnostic criteria, inclusion criteria, and exclusion criteria, the receptors who are eligible will be the participants. Once the patients qualified and agreed to participate in the study, informed consent will be obtained before running the series of baseline measurement assessments. All participants will provide written informed consent. Every participant will be assigned a unique study identification number, which is the only identifier included on study paper form and database. We promised that all records will remain secure and any possible harm from the trial will be provided free of medical treatment by the hospitals. The potential participants can consult with doctors regarding any information about the study details including the study purpose, procedures, benefits, and potential risks.

Ethics approval was obtained from the Clinical Trial Ethics Committee of The Third Affiliated Hospital of Zhejiang Chinese Medical University (NO. ZSLL-KY-2017-033) and Jiaxing Hospital of Traditional Chinese Medicine (NO. 2018-JZLK-002). The protocol version 2.0 was approved in December 2017. The results will be disseminated by telephone during follow-up calls and presentation at peer-reviewed journals. This trial will be conducted in accordance with the Declaration of Helsinki.

#### 2.2.3 Random grouping, blind design, and implementation

The random grouping operation is carried out by using Dynamic Randomization Method of Central Stochastic System (network edition). For the study on the acupuncture treatment, the doctors have to contact the patient directly. It is difficult for acupuncturists to conduct double-blind trials in clinic. However, the other participants, including patients, observation index recorders, and data statisticians, will be implemented blind. They are not aware of the groups of the subjects. At the same time, the subjects will be evaluated by blinding effect.

To ensure the better implementation of the blind, the study will provide separate treatment as far as possible for each subject. This measure can effectively avoid communication between patients receiving different treatments, thereby improving the reliability of clinical trial.

### 2.3 Participants population

It is expected that 120 participants will be recruited in the Third Affiliated Hospital of Zhejiang Chinese Medical University and Jiaxing Hospital of Traditional Chinese Medicine.

#### 2.3.1 Diagnostic criteria

The diagnostic criteria are based on *The International Classification of Headache Disorders, 3rd Edition (Beta Version)*^[23]^ published by the Headache Classification Committee of the International Headache Society (IHS) in 2013.

1. Pain on one side of the face should be conformed to the following criteria 2 and 3, with a paroxysm of at least 3 times.
2. Occurring in ≥1 divisions of the trigeminal nerve, with no radiation beyond the trigeminal distribution.
3. Characteristics of pain contain at least 3 of the following 4 characteristics:
  a. Recurring in paroxysmal attacks lasting from a fraction of a second to 2 minutes.
  b. Severe intensity.
  c. Electric shock-like, shooting, stabbing in quality.
  d. Precipitated by innocuous stimuli to the affected side of the face.
4. No neurological impairment.
5. Eliminating other disease that would cause the pain.

#### 2.3.2 Inclusion criteria

1. Paroxysm pain involving branches of the 2nd and/or 3rd branch of trigeminal nerve.
2. The VAS baseline score ≥5 points, have an attack >3 times a day, at least 4 days a week.
3. 18 years ≤ age ≤ 80 years.
4. Clear consciousness, have the ability of pain perception and resolution, can complete the basic communication.
5. Signed informed consent and volunteered to participate in this study.

#### 2.3.3 Exclusion criteria

1. Those patients with epilepsy, head injury, or other related neurological diseases.
2. Patients with serious heart, liver, kidney damage or cognitive impairment, aphasia, mental disorders, or unable to cooperate with the treatment.
3. Combined with hypertension but poor control.
4. Severe depressive with definitive diagnosis recently.
5. Pregnant and lactating patients.
6. Installing pacemakers.
7. For any other reason that is not suitable for the treatment of EA.

#### 2.3.4 Removed criteria

The subjects who have entered a group of research but who met one of the following conditions should be removed:

1. Those who do not meet exactly with the inclusion and exclusion criteria of the trial.
2. There are obviously adverse reactions during the treatment.
3. The subjects are not treated according to the treatment plan of the group after they have entered.

The removed cases should indicate the reason and retain the original medical records. No participation of statistical analysis of efficacy, but those who have received at least 1 treatment can participate in analysis of adverse reaction.

### 2.4 Intervention procedures

The acupuncture needles of the study will be unified with HuaTuo brand disposable acupuncture needles produced by Suzhou Medical Products Factory CO., LTD., and the specifications are ϕ0.18 × 25 mm and ϕ0.25 × 40 mm. The EA instrument is unified by the acupoint neural stimulator HuaTuo SDZ-IIB, which was produced by SuZhou Medical Supplies Co., Ltd.

#### 2.4.1 EA + carbamazepine group

##### 2.4.1.1 Acupuncture acupoints

Local acupoints:

Main acupoints: The affected side of Si-bai (ST2), Xia-guan (ST7), Di-cang (ST4).
Adjunct acupoints: Quan-liao (SI18) for pain in the 2th branch, Jia-che (ST6) for pain in the 3^rd^ branch, and A-shi-xue (2 acupoints will be selected from the second branch or the third one).
Distal acupoints: The 2 sides of He-gu (LI4) and Wai-guan (TE5).

##### 2.4.1.2 The position of acupoints

According to the 2006 People's Republic of China National Standard (GB/T 12346-2006) “Acupoints names and positioning,” the position of acupoints is direct in Table 2.

**Table 2:**
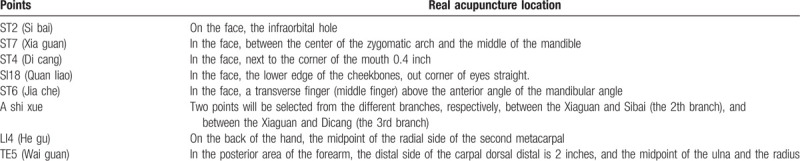
Acupuncture acupoints locations.

##### 2.4.1.3 Manipulation

Needling operation: Subjects with supine position, general relaxation, routine disinfection of the facial skin. The superficial acupuncture will be applied for local points. The tiny needles (0.18 × 25 mm) will be selected to stimulate the local points with shallow row needling according to the distribution of neuropathy branch of trigeminal neuralgia. The operation of acupuncture should be gentle and the depth of acupuncture should be within 5 mm. It is not required a sense of “Qi” and should not touch the trigger point (the trigger points are the pain sensitive places that light touch can trigger severe pain, such as tiff, cheek, nose and so on. The doctor should have a communication with the subject before needling to clear the position of the trigger (do not needle or touch it). The He-gu (LI4) and Wai-guan (SJ5) points will be selected as the distal points stimulating with 0.25 × 40 mm needle (20–30 mm depth). After the subject has a sense of acid distention (“get Qi”), the reducing method of twirling and lifting-inserting will be applied 10 times. If the subject is feeling pain seriously, the distal acupoints can be needling firstly with reducing method sharply until the pain relief. Then it is turn for needling the local acupoints.

EA parameters: The adjunct acupoints of (ST7 and SL18) or (ST7 and ST6) will be received EA treatment by HuaTuo SDZ-IIB acupoint neural stimulator. Also, the distal acupoints of LI4 and SJ5 will be received EA treatment. The EA parameter is a dilute wave with 2/100 Hz, the treatment time is 60 minutes, and the current intensity is comfortable to subjects.

##### 2.4.1.4 Oral carbamazepine

Carbamazepine tablets (Jiangsu Pengli Pharmaceutical CO., LTD., 0.1 g × 100 tablets) should be took orally, 0.1 g each time, thrice daily.

##### 2.4.1.5 Frequency, duration, and time

Treatment will be performed 3 times per week, and 4 weeks of continuous interventions for a total of 12 times.

#### 2.4.2 EA + placebo group

In this group, patients will receive EA treatment, and the operation of EA is the same as that in the EA + carbamazepine group. Meanwhile, patients will also take the placebo with the same appearance and size as carbamazepine, 0.1 g of each dose and three times a day.

#### 2.4.3 Sham EA + carbamazepine group

Selection of points and locations: the nonmeridional points which are means to the points beside 5 to 10 mm of the real acupoints (avoid the trigger point) in the EA group will be selected and needled with more shallow acupuncture (the depth of needling is about 1–2 mm).

The operation of shame EA: The HuaTuo SDZ-IIB acupoint neural stimulator with damaged electrode wires will be selected to connect the points next to the Xia-guan (ST7) and Quan-liao (SI18), He-gu (LI4), and Wai-guan (TE5). The frequency, intensity, and retaining time will be same as EA group. The subjects can see the display screen and parameter settings of stimulator; however, there is no electricity output in fact.

The dosage and frequency of oral carbamazepine tablets are same as above part.

#### 2.4.4 Sham EA + placebo group

In this group, patients will receive sham EA treatment, the operation of sham EA is the same as that in the sham EA + carbamazepine group. Patients will also take the placebo with the same appearance and size as carbamazepine, 0.1 g of each dose and three times a day.

### 2.5 Emergency treatment

It is forbidden to use another analgesic therapy at any time during the trial. Unless the patients unable to relieve pain continually and its seriously affects patient's daily work and life, and they can urgently use carbamazepine 0.2 g once daily and take it after a meal. The use date and time and dosage of emergency drugs or other analgesic measures must be recorded in time.

### 2.6 Outcome measures

Participants are required to write a verifiable diary card to record their pain symptoms in 24 hours every day. The diary includes the following items: daily attack frequency (spontaneous and provoking pain frequency); provoking factors; average pain intensity (VAS) in the past 24 hours; side effect in the past 24 hours (including side effect of drugs or acupuncture); another analgesia therapies been adopted (please record it if any).

#### 2.6.1 Indexes of main outcome evaluation

1. Change from baseline intensity of pain at 6 months (evaluation of the pain by VAS with 0–10 points).
2. Change from baseline brief introduction of 2-week pain.

Evaluation method: According to pain diary, we will take average data of 2 weeks before treatment (−2∼0 week) as baseline, 2 weeks of treatment phase (1∼2 week) as data of treatment phase I, 4 weeks of treatment phase (3∼4 week) as data of treatment phase II. During the follow-up period, the pain diary card will be continued in the 2 weeks before 3 months (11∼12 week)/6 months (23∼24 week) after treatments.

#### 2.6.2 Indexes of secondary outcome evaluation

1. Brief Pain Inventory-Facial scale (BPI-Facial)
2. Patient Global Impression of Change (PGIC)
3. Short-Form McGill Pain Questionnaire (SF-MPQ)
4. Short- Form 36 Questionnaire (SF-36)

#### 2.6.3 Research cycle and evaluation time points

The treatment period of this research is 4 weeks, and the follow-up period is 6 months. The above indexes are observed at the time points of baseline (the same day with the grouping and treatment for the subject), treatment period I (the end of the 2nd week), treatment period II (the end of the 4th week), follow-up period I (3 months after treatment), and follow-up period II (6th months after treatment). The main outcome indexes will be measured weekly (1–4 weeks) during the treatment period.

### 2.7 Safety and blindness evaluation

The abnormal acupuncture should be record, including broken needle, left needle, fainting, unbearable acupuncture pain (VAS ≥8 points), local hematoma, infection, and abscess. The adverse reaction of carbamazepine should be recorded, including blurred or diplopia vision, drowsiness, weakness, nausea, vomiting, mental, and/or neurological abnormalities and others.

The participants will be asked to accept true acupuncture, sham acupuncture, or uncertainty at the end of the 4th week of any acupuncture treatment. Researchers will compare the percentages of the 2 groups.

### 2.8 Quality control and quality assurance

The operators of the centers for specific acupuncture treatment must have the qualifications of the acupuncturist and are independent of the clinical treatment for >2 years. To ensure the smooth progress of the research, the first month before the clinical trial is officially launched, the research team will convene and complete the special clinical training, and conduct unified training for the clinical researchers of the 2 centers. Key train is carried out on that implementation of the subject and the Standard Operation Procedure (SOP) and gauge evaluation so that each clinical researcher is familiar with the research process and detailed implementation rules to improve the internal observation consistency of the researcher and the consistency between the observers, to ensure the reliability of the clinical study conclusion.

All observations and findings in clinical studies should be verified and confirmed repeatedly to ensure the reliability and authenticity of the data and to ensure that the results and conclusions of clinical research are derived from the original data.

The appointment of specialized personnel for experimental data collection and statistics will control the test bias.

The quality control measures of the centers will be implemented through regular monitoring of the two centers.

The compliance of patients was measured by the number of treatments. The calculation formula is as follows:

Criteria are the number of treatments that the subject has received >80% of the treatment (including 80%).

### 2.9 Statistical analysis plan

#### 2.9.1 Data collection, entry, and storage

The Case Report Form (CRF) will be first completed on paper and then entered into the randomized controlled experimental system by 2 independent investigators to act as the first level of control to ensure the accuracy of the data. If any omission/review or blurring is found, check the gauge in time and verify the quality of the recovery gauge strictly.

The special person is responsible for the management of various documents, and there are special folders for storage in dedicated files, so that the test researcher can view it, and have access and access records. The original CRFs and all other forms (including the consent forms) will be archived securely at The Third Affiliated Hospital of Zhejiang Chinese Medical University for 5 years following publication of the last paper or report from the study.

#### 2.9.2 Statistical processing

The final trial data set will be under the custody of The Third Affiliated Hospital of Zhejiang Chinese Medical University and the manager of the Clinical Evaluation Center of Zhejiang Provincial Hospital of TCM. The safety of the study will be monitored by a Data and Safety Monitoring Board (DSMB) of the Clinical Evaluation Center of Zhejiang Provincial Hospital of TCM, which consists of independent clinical experts and statisticians with access to unblinded data.

All statistical analyses will be analyzed by the SAS 9.3 software analysis (SAS Institute, Cary, NC). The main analysis contents include: 4 groups of cases distribution which demonstrate the total loss rate due to adverse events; compare demographic data and other baseline values to measure the comparability of the four groups; compliance analysis of the 4 groups according to the amount of time to receive the appropriate treatment; effectiveness analysis according to the therapeutic indexes; effect factor analysis including age, sex, duration, combined medication, and other factors on the efficacy; safety analysis according to the number of cases of adverse events.

All the statistical tests were double-sided, and the *P* value is ≤.05 and the difference is statistically significant.

### 2.10 Patient and public involvement

Patients and the public were not involved in the design of the study. However, patients and public will be involved in the recruitment by disseminating the information about the trial with public communications. Patients will be involved in the results presentation partly throughout the record of pain diary, which is a feedback of brief introduction of pain.

## 3 Discussion

The current conventional treatment on the CTN, such as drugs, surgery, and other adjuvant methods, may provide some modest relief from pain.^[7,24]^ However, for CTN is characterized by intractability, patients have poor compliance with medications and surgical treatments as they cause more adverse reactions, and the treatments also can entail significant cost to patients and society.^[25]^ In traditional Chinese medicine (TCM), the trigeminal neuralgia belongs to the category of facial pain, which has been discussed in TCM ancient literatures for thousands of years. TCM has a long history of treating the disease, especially acupuncture has a significant effect on it. Acupuncture is widely applied and is researched in complementary and alternative medicine to relieve pain in recent years.^[26–28]^ Although no systematic research has been carried out and there is no conclusive evidence of acupuncture therapy to trigeminal neuralgia in evidence-based medicine. This study intends to systematically study the effects of acupuncture combined with EA on the CTN under the RCT clinical research paradigm to help filling in that blank.

The establishment of an effective treatment program of EA therapy for CTN is the first strengthen and key technique in the study. The therapy technologies include acupoints selection, needing technique, EA parameters selection, and how to apply acupuncture combined with carbamazepine. The intervention adopted in this study is developed based on profound TCM theory and supported by comprehensive scientific research. The theory guiding the acupoints selection and acupuncture manipulation parameter has been studied, identified, and well-demonstrated through clinical work.^[29,30]^ Specifically, the mechanism was explored on how EA can relieve pain. On the one hand, EA can increase the release of inhibitory neurotransmitters through the corresponding receptors in the nerve cells, thereby inhibiting the generation and conduction of abnormal discharges. On the other hand, EA can alleviate the demyelination of nerve roots caused by compression, and improve the condition in which the axons are excessively tightly aligned.^[21,31–33]^ Above all, the treatment program in the study is clinically active, what should we do in the study is demonstrating then to be optimization to popularize.

The trial design and the quality control of the study are other strengths of this clinical project. Grasping the above key points will help to develop a scientific and standardized research program, which can provide a higher level of evidence-based medical evidence for the clinical effect of CTN by EA therapy. In design of this study, according to international authoritative clinical research methods, the grouping in RCT study is very important. Effective grouping can reflect multiple problems from a limited number of cases. Because of the characteristics of CTN, severe pain, and relatively low incidence, we have set up a total of 4 groups: EA + carbamazepine group, sham EA + carbamazepine group, EA + placebo group, and sham EA + placebo group. Through a pair-wise comparison, the therapeutic effects of EA and carbamazepine can be clarified, and it can be judged whether EA has the advantage of curative effect and whether EA and carbamazepine combined have better efficacy than EA alone. In addition, the placebo effect was attached importance to the outcomes. Researchers have recognized the placebo effect, in which the illusion of treatment, such as pills without an active ingredient, produces actual medical benefits.^[34,35]^ Accurate effect of drugs is confusing; therefore, we set up a placebo as a control group in this study so that we can compare the effects of EA, carbamazepine, and EA combined with carbamazepine. With participants unknowing of their intervention assignments after randomization, this ensures the study will be implemented in accordance with the daily clinical practices to the most extent; thus, the external validity can be mostly warranted.

However, due to the low incidence of CTN and the difficulty in case collection, the small sample size became the main limitation of the study. Compared with the large samples in multicenter trials recently years, this trial may not be strengthened. Nevertheless, the participants’ recruitment of sample size in the trial will be guaranteed. The quality control of the clinical study will be carried out in the trial. With the strength of ingenious grouping, the judgment from clinical experiences and relevant findings from previous studies can help in identifying and justifying the effect with clinical significance. Therefore, the information bias due to lack of samples could be controlled to a considerable extent.

## 4 Conclusion

The trial design is a 2-center, randomized, sham-controlled, placebo-controlled clinical study to compare the effectiveness and safety of EA with carbamazepine for CTN. The outcomes will help to provide a higher level of evidence-based medical evidence for the clinical treatment of CTN by EA therapy.

### 4.1 Trial status

The time of the first patient enrolment was July 12, 2018. The trial is recruiting participants now.

## Acknowledgments

The authors express their profound appreciation to all coordinators, therapists, and evaluators for their diligence. They thank all of the patients who participated in the study.

## Author contributions

Jianqiao Fang is responsible for this study. Lifang Chen, Yi Liang and Jing Sun designed the trial. Jing Sun, Rongrong Li, Xiaoyu Li and Hantong Hu write the manuscript. Rongrong Li, Xiaoyu Li, Qifei Zhang and Ruohan Sun participate in recruitment and administration. Yi Liang and Xiaomei Shao planned a data analysis solution. All the authors have read, revised and approved this version of manuscript.
